# Controls on the Isotopic Composition of Nitrite (δ^15^N and δ^18^O) during Denitrification in Freshwater Sediments

**DOI:** 10.1038/s41598-019-54014-3

**Published:** 2019-12-16

**Authors:** Mathieu Sebilo, Giovanni Aloisi, Bernhard Mayer, Emilie Perrin, Véronique Vaury, Aurélie Mothet, Anniet M. Laverman

**Affiliations:** 1 Sorbonne Université, CNRS, IEES, F-75005 Paris, France; 2 CNRS/UNIV PAU & PAYS ADOUR/E2S UPPA, Institut des Sciences Analytiques et de Physicochimie pour l’Environnement et les Matériaux (IPREM), UMR 5254, 64000 Pau, France; 3 Université de Paris, Institut de physique du globe de Paris, CNRS, 1 Rue Jussieu, 75005 Paris, France; 4 Applied Geochemistry Group, Department of Geoscience, University of Calgary, 2500 University Drive NW, Calgary, Alberta Canada T2N 1N4; 5 Université de Rennes 1, CNRS, Ecobio, campus de Beaulieu, 263 avenue du Général Leclerc, 35042 Rennes Cédex, France

**Keywords:** Element cycles, Element cycles

## Abstract

The microbial reduction of nitrate, via nitrite into gaseous di-nitrogen (denitrification) plays a major role in nitrogen removal from aquatic ecosystems. Natural abundance stable isotope measurements can reveal insights into the dynamics of production and consumption of nitrite during denitrification. In this study, batch experiments with environmental bacterial communities were used to investigate variations of concentrations and isotope compositions of both nitrite and nitrate under anoxic conditions. To this end, denitrification experiments were carried out with nitrite or nitrate as sole electron acceptors at two substrate levels respectively. For experiments with nitrate as substrate, where the intermediate compound nitrite is both substrate and product of denitrification, calculations of the extent of isotope fractionation were conducted using a non-steady state model capable of tracing chemical and isotope kinetics during denitrification. This study showed that nitrogen isotope fractionation was lower during the use of nitrite as substrate (ε = −4.2 and −4.5‰ for both treatments) as compared to experiments where nitrite was produced as an intermediate during nitrate reduction (ε = −10 and −15‰ for both treatments). This discrepancy might be due to isotopic fractionation within the membrane of denitrifiers. Moreover, our results confirmed previously observed rapid biotic oxygen isotope exchange between nitrite and water.

## Introduction

The growing world population requires an increased food production, necessitating the intensive use of nitrogen-containing synthetic fertilizers, which has led to nitrate pollution in many surface water and groundwater bodies^1–5^. Even though essential for plants, dissolved nitrate can be responsible for contamination of drinking water supplies from surface and groundwaters, posing a potential threat to human health^6–10^. Fortunately, it has been shown that denitrification plays a significant role in the mitigation of nitrate pollution^11–13^. Denitrification is the microbial dissimilatory reduction of NO_3_^−^ to N_2_ via several steps, governed by different enzymes^14–16^. During this beneficial removal of NO_3_^−^, the intermediates nitrite (NO_2_^−^), a toxic component at low concentrations^17^, and nitrous oxide (N_2_O), which participates in ozone layer destruction^18^, are formed. Whereas the production of N_2_O has received considerable attention due to its role as a potent greenhouse gas, the intermediate compound nitrite (NO_2_^−^) has received much less attention since it is often assumed to be thermodynamically unstable, short-lived, and therefore not accumulating in the environment. However, nitrite has been detected at concentrations exceeding the European Water Framework Directive (EU WFD) of 0.009 mg N L^−1^ in urbanized rivers^19–23^. Therefore, a better understanding of its dynamics in aquatic systems is highly desirable.

**Figure 1:**
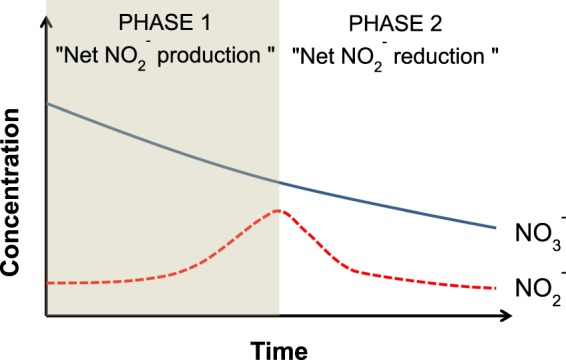
Theoretical trends of NO_3_^−^ and NO_2_^−^ concentrations during denitrification.

During denitrification, nitrite is both the product of nitrate reduction and the substrate of its own reduction into NO, N_2_O and N_2_ (Fig. 1):

1 $${{\rm{NO}}}_{3}^{-}+2{{\rm{e}}}^{-}+2{{\rm{H}}}^{+}\to {{\rm{NO}}}_{2}^{-}+{{\rm{H}}}_{2}{\rm{O}}$$

2 $${{\rm{NO}}}_{2}^{-}+{{\rm{e}}}^{-}+2{{\rm{H}}}^{+}\to {\rm{NO}}+{{\rm{H}}}_{2}{\rm{O}}$$

3 $$2{\rm{NO}}+2{{\rm{e}}}^{-}+2{{\rm{H}}}^{+}\to {{\rm{N}}}_{2}{\rm{O}}+{{\rm{H}}}_{2}{\rm{O}}$$

4 $${{\rm{N}}}_{2}{\rm{O}}+2{{\rm{e}}}^{-}+2{{\rm{H}}}^{+}\to {{\rm{N}}}_{2}+{{\rm{H}}}_{2}{\rm{O}}$$

Nitrate reduction initially results in nitrite production. During phase 1, the “net nitrite production” phase, the NO_2_^−^ concentration rises and reaches a maximum; then, in phase 2, the concentration of nitrite decreases until nitrite is completely consumed. It is noted that during phase 1 production as well as reduction of NO_2_^−^ can take place simultaneously. The same holds for phase 2, where nitrite is mostly reduced but also still being produced. In addition of being an intermediate during denitrification, nitrite is also an important intermediate at the crossroads of other nitrogen transformation processes, such as codenitrification^24,25^, dissimilatory nitrate reduction to ammonium (DNRA)^26–28^, anaerobic ammonium oxidation (Anammox)^29^, nitrifier denitrification^30^ and nitrification^31,32^.

Stable isotope techniques can provide unique insights into sources and processes regulating the N cycle in ecosystems. The nitrogen and oxygen isotope ratios of NO_3_^−^ (δ^15^N and δ^18^O) have been used since several decades to better distinguish the origin of NO_3_^− ^^33–36^. In addition, it provides information regarding the processes involved in its transformation, *e.g*. denitrification and nitrification in lakes, groundwater, riparian zones, rivers, soils, and marine environments^37–43^. Denitrification is accompanied by significant N and O isotope fractionation affecting the remaining NO_3_^−^ due to reaction kinetics differences between the lighter and the heavier isotopes of N and O (^14^N vs. ^15^N and ^16^O vs. ^18^O). Enrichment in heavy isotopes of N and O in the remaining nitrate is caused by the fact that ^14^N and ^16^O react faster than ^15^N and ^18^O during denitrification^33,44^.

Denitrification is a unidirectional reaction in which nitrate is reduced to gaseous nitrogen (N_2_) via different intermediates (NO_2_^−^, NO, and N_2_O). The N and O isotope enrichment factors ε^15^N and ε^18^O during denitrification are usually determined by measuring variations in concentrations and isotope ratios of the remaining nitrate, while the isotopic compositions of the final product N_2_ or of the intermediate compounds such as NO_2_^−^ and N_2_O are rarely analyzed. In past studies, the concentrations and isotopic compositions of nitrate and nitrite have not always been separately determined due to analytical limitations for the latter. Therefore, the “net ε^15^N” and “net ε^18^O” determined for the entire denitrification process assessed solely based on measurements of the progressively disappearing nitrate may not always be accurate.

Only a few studies used nitrite (NO_2_^−^) rather than nitrate as source of nitrogen for the determination of ε^15^N for nitrite reduction. Two of these studies were carried out in soil experiments^45,46^ another with a microbial culture^43^. Due to new chemical reduction analytical methods, it is now possible to determine the isotopic composition of NO_2_^−^ alone, separating the isotopic analyses of NO_3_^−^ from those of coexisting NO_2_^− ^^47–50^. Recent studies of the isotopic composition of nitrite focused mainly on nitrification^51–56^. A few studies mention ε^15^N values for nitrite reduction during denitrification. These studies were conducted using pure cultures of denitrifiers^41–43^, marine incubations^42^, *in-situ* marine environments^55^ or more recently abiotic denitrification^57^. In order to improve our knowledge on the isotope effects affecting nitrite, we investigated isotope fractionation during denitrification in laboratory experiments with environmental bacterial communities. Nitrite was either applied as a substrate or analyzed as intermediate during nitrate reduction.

Since during denitrification and nitrate consumption nitrite is both substrate and product, it is not possible to use the Rayleigh equation to calculate concentrations and isotope compositions of the intermediate compound nitrite. For this reason, we developed the *Isonitrite* numerical model that simulates the evolution of oxygen and nitrogen isotope compositions of NO_3_^−^ and NO_2_^−^ during simultaneous nitrite production and consumption. The model, constrained by our experimental data, is used to estimate isotope fractionation factors of oxygen and nitrogen (ε^15^N and ε^18^O) associated to denitrification. To our knowledge, this study is the first focusing on freshwater benthic denitrifying communities with two distinct sources of dissolved inorganic nitrogen, *i.e*, nitrite or nitrate.

## Results

### Isotopic enrichment factors associated with nitrite reduction

**Figure 2:**
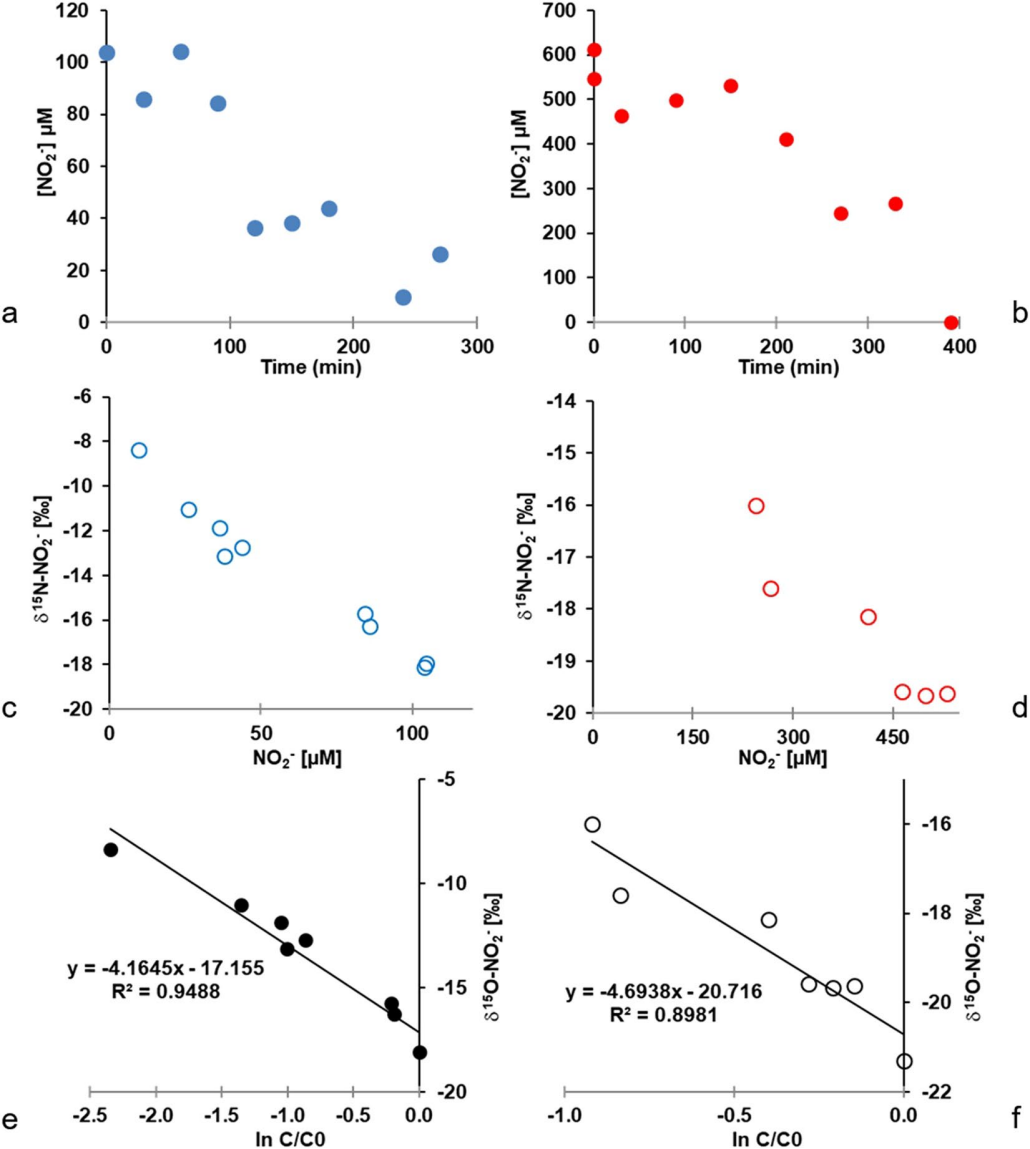
Kinetics of nitrite concentrations (**a**,**b**), variations of δ^15^N (**c**,**d**) and calculation of ε^15^N (**e**,**f**) for low and high nitrite treatments respectively.

In the low nitrite experiment (101 µM), concentrations decreased with time reaching 9.7 µM after 240 min (Fig. 2a). In the high nitrite experiment (614 µM), concentrations decreased continuously to 0 µM after 390 min (Fig. 2b). Nitrite reduction rates of 119 nmol min^−1^ g^−1^ were observed at high initial nitrite concentrations whereas the low initial nitrite concentration resulted in lower nitrite reduction rates of 35 nmol min^−1^ g^−1^ under the experimental conditions.

The decrease in nitrite concentrations was associated with a change in δ^15^N-NO_2_^−^ of the remaining NO_2_^−^ increasing from −18.1‰ to −8.4‰ and from −19.6‰ to −17.6‰ for low and high treatments respectively (Fig. 2c,d). The N isotope enrichment factors associated with the reduction of NO_2_^−^ was −4.2 and −4.7‰ for low and high nitrite treatments respectively (Fig. 2e,f).

**Figure 3:**
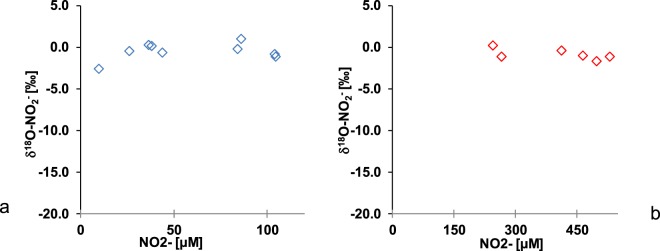
Variations of δ^18^O-NO_2_^−^ with the decrease of nitrite concentration for low (**a**) and high (**b**) nitrite treatments.

The reduction of nitrite generated constant and low oxygen isotope values in the remaining nitrite (Fig. 3a,b). The oxygen isotope ratios of nitrite remained constant throughout the experiment with mean δ^18^O-NO_2_^−^ of −0.6 ± 1.1‰ and −0.9 ± 0.6‰ for low and high nitrite treatments respectively.

### Isotopic enrichment factor associated with nitrate reduction

**Figure 4:**
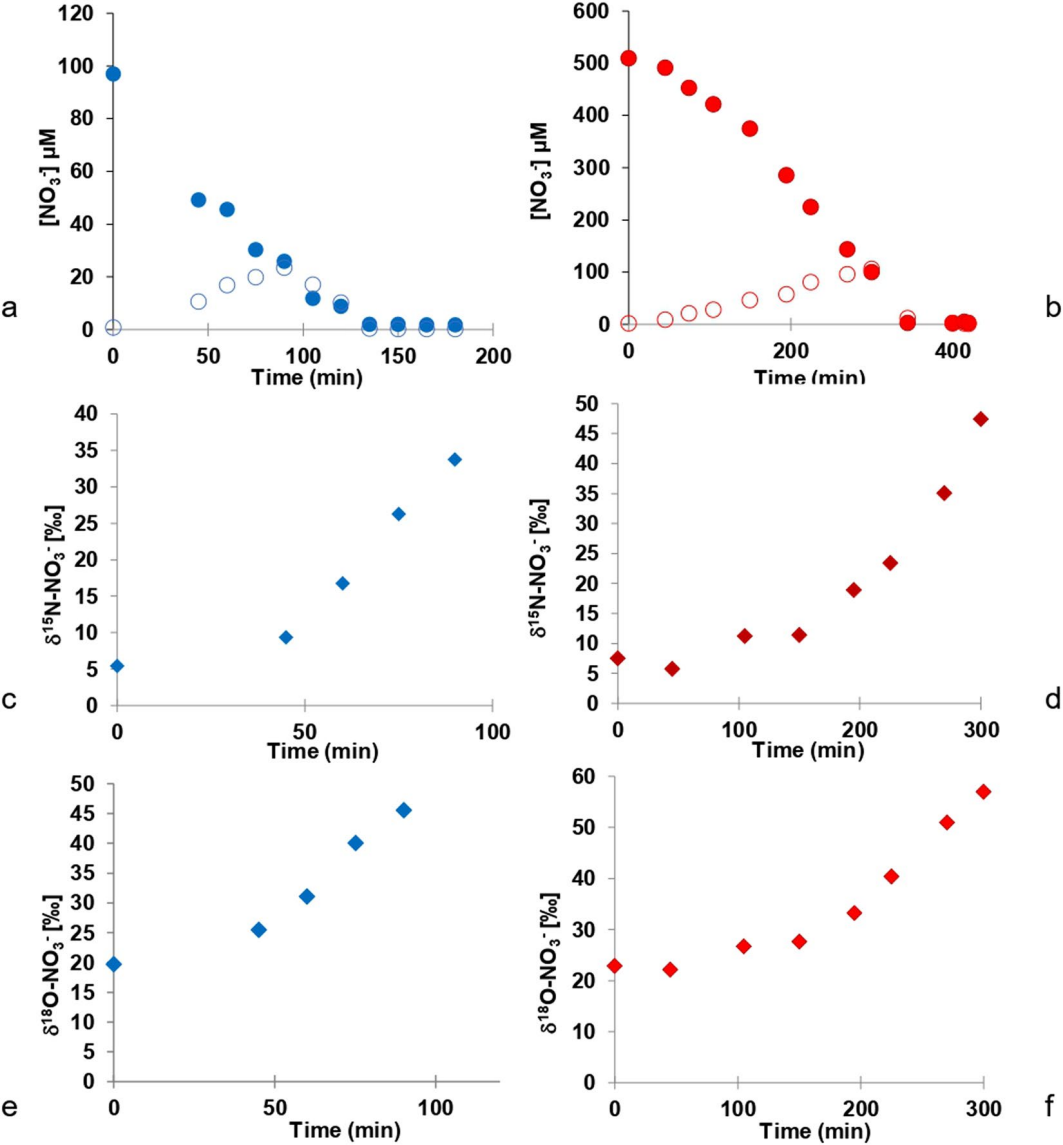
Variations of nitrite (open symbol) and nitrate (filled symbol) concentrations (**a,b**), variations of δ^15^N-NO_3_^−^ (**c**,**d**) and of δ^18^O-NO_3_^−^ (**e**,**f**) for low and high nitrate treatments respectively.

Similar to the experiments carried out with nitrite only, the kinetics of denitrification were investigated with solutions with low (97 µM) and high (508 µM) initial nitrate concentrations (Fig. 4b). Nitrate concentrations decreased from 97 to 1.8 µM and from 508 to 1.2 µM over the 180 and 420 minutes of the experiments. The rates of nitrate reduction were 50 nmol g^−1^ min^−1^ and 137 nmol g^−1^ min^−1^ for low and high nitrate treatments respectively. The nitrate reduction rates are in the same range as the nitrite reduction rates (35 and 119 nmol g^−1^ min^−1^, see previous section).

In both nitrate experiments (low and high), initial NO_2_^−^ concentrations were less than 1 µM. In the low nitrate treatment, NO_2_^−^ concentrations increased to 23 µM at 90 minutes and then decreased to 0.2 µM at 105 minutes (Fig. 4a, open symbols). For the high nitrate treatment, NO_2_^−^ concentrations increased to 105 µM at 300 minutes and subsequently decreased to 0.4 µM at 420 minutes (Fig. 4b, open symbols). As expected, the trends in NO_2_^−^ concentrations reveal two phases; an increase due to nitrate reduction to nitrite leading to an initial net production of nitrite, followed by a decrease of nitrite concentrations as the newly formed nitrite is further reduced to N_2_ (see Fig. 1).

In the two experiments with NO_3_^−^, the decrease in NO_3_^−^ concentrations was accompanied by a corresponding enrichment of ^15^N in the remaining NO_3_^−^. The δ^15^N-NO_3_^−^ of the remaining nitrate increased from 5.5 to 33.7‰ within 90 minutes and from 7.5 to 47.4‰ within 300 minutes in low and high nitrate treatments (Fig. 4c,d). The decrease in NO_3_^−^ concentrations due to denitrification was also accompanied by a corresponding enrichment of ^18^O in the remaining NO_3_^−^. δ^18^O-NO_3_^−^ increased from 19.8 to 45.6‰ within 90 minutes and from 22.9 to 57.0‰ within 300 minutes in low and high nitrate treatments respectively (Fig. 4e,f). The increase in δ^18^O-NO_3_^−^ was initially very slow as long as nitrite was net produced, in all likelihood due to the rapid oxygen isotope exchange between nitrite and water.

**Figure 5:**
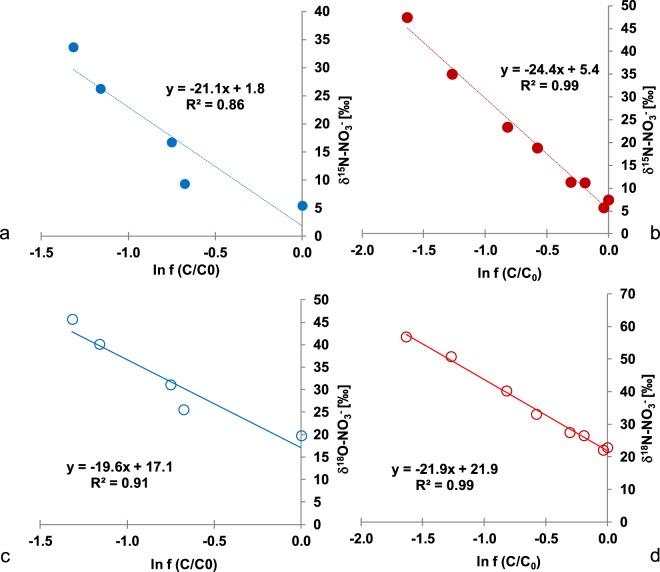
Calculation of ε^15^N-NO_3_^−^ (**a**,**b**) and ε^18^O-NO_3_^−^ (**c**,**d**) for low and high nitrate treatments respectively.

The N isotope enrichment factor associated with the reduction of nitrate varied between −21.1 and −24.4‰ (Fig. 5a,b) and the O isotope enrichment factors ranged between −19.6 and −21.9‰ (Fig. 5c,d) for low and high nitrate treatments respectively. Despite different nitrate reduction rates between the two experimental treatments with amended nitrate concentrations, the observed N and O isotopic enrichment factors did not vary by more than 3.3‰.

### Modeling kinetics and isotopic composition of nitrite and nitrate during denitrification

**Figure 6:**
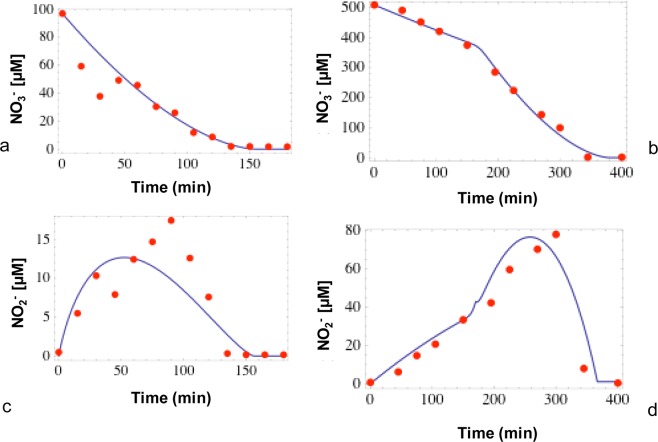
Measured (red dots) and modeled (blue lines) nitrate (**a**,**b**) and nitrite concentrations (**c**,**d**) for low and high nitrate treatments respectively.

**Figure 7:**
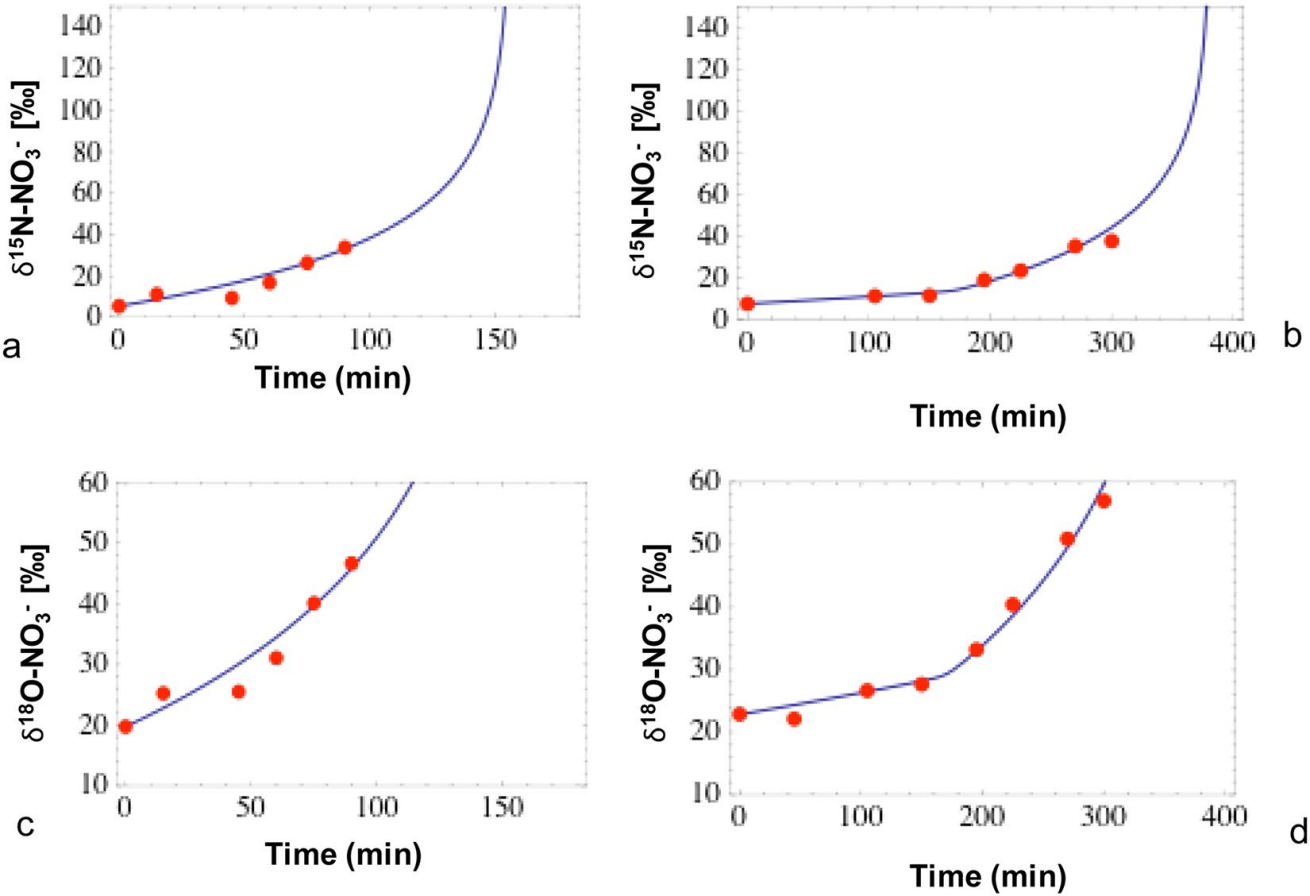
Measured (red dots) and modeled (blue lines) δ^15^N-NO_3_^−^ (**a**,**b**) and δ^18^O-NO_3_^−^ (**c**,**d**) for low and high nitrate treatments respectively.

The Isonitrite model provides the kinetic parameters of the multi-step process of NO_3_^−^ reduction to N_2_ via the intermediate NO_2_^−^. The model was applied to simulate concentrations and isotope compositions of nitrite and nitrate observed in the batch experiments for the low and high concentration treatments. Overall, the model achieved good simulations of the temporal evolution of NO_3_^−^ and NO_2_^−^ concentrations (Fig. 6) and N and O isotope ratios of nitrate and nitrite (Fig. 7).

**Figure 8:**
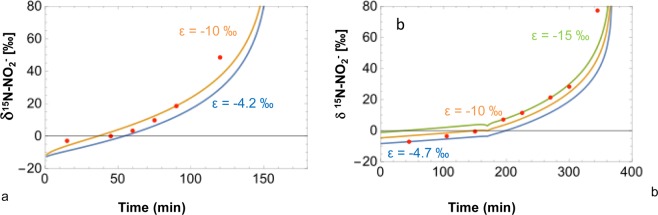
Determination of ε^15^N of nitrite during the reduction of nitrate for low (**a**) and high (**b**) nitrate treatment.

When simulating the low-nitrate treatment, the model fit of the experimental NO_3_^−^ and NO_2_^−^ concentrations was obtained by using kinetic constants of 4.2 × 10^−5^ min^−1^ for NO_3_^−^ reduction (equation 11 in Methods) and 7.2 × 10^−5^ min^−1^ for NO_2_^−^ reduction (Eq. 8), and a reaction order (*n* in Eqs. 7 and 8) of 0.4. In these experiments, the model constrains N and O isotope enrichment factors (ε^15^N = −19‰, ε^18^O = −18‰ for NO_3_^−^ reduction) that are very close to the values obtained by applying the Rayleigh model to the data (Fig. 5). This indicates that the model is able to correctly reproduce the N and O isotope fractionation of the reduction of NO_3_^−^ to NO_2_^−^. However, in order to obtain a good fit of the model to the concentration and isotope data obtained in the multi-step (NO_3_^−^ → NO_2_^−^ → N_2_) experiment (Fig. 8a), it was necessary to impose a higher ε^15^Ν for NO_2_^−^ reduction (−10‰) than the value determined in batch experiments where only NO_2_^−^ reduction occurred (−4.2‰; Fig. 2).

Similar results were obtained with the model applied to the experiment with high nitrate treatment. However, to obtain a good fit of the NO_3_^−^ and NO_2_^−^ concentration trends, a slightly different approach was implemented to simulate the kinetics of NO_3_^−^ and NO_2_^−^ reduction. To reproduce the increase in NO_3_^−^ consumption at 170 minutes observed in this experiment (Fig. 5c), a change in the kinetic constant of NO_3_ reduction from 1.8 × 10^−5^ min^−1^ to 6.9 × 10^−5^ min^−1^ at 170 minutes was necessary. Also, the NO_2_^−^ reduction rate was set to 6 × 10^−1^ µM min^−1^ for the first 170 minutes, and to 1.8 × 10^−2^ µM min^−1^ for the remaining part of the experimental high nitrate treatment to achieve a satisfactory fit of the observed nitrite concentrations (Fig. 6d). A very good fit of the experimental trends in δ^15^N-NO_3_^−^ and δ^18^O-NO_3_^−^ was obtained by imposing model N and O isotope enrichment factors (ε^15^Ν = −19.1‰, ε^18^O = −19.2‰ for NO_3_^−^ reduction) that were very close to the values measured in the batch experiments where only NO_3_^−^ was reduced (Fig. 7). Similar to the low nitrate experiment, it was necessary to use a higher model ε^15^Ν for NO_2_^−^ reduction (ε varied between −10 and −15‰) than that determined in batch experiments where only NO_2_^−^ reduction took place (ε = −4.7‰) to obtain a good model fit for the data of the multi-step NO_3_^−^ → NO_2_^−^ → N_2_ experiment (Fig. 8b). This confirms that the Isonitrite model is capable of accurately predicting the concentrations of nitrate and nitrite, and the isotope composition of the remaining nitrate during denitrification.

## Discussion

### N isotope fractionation during denitrification and transformation of nitrite and nitrate

Even though initial nitrite concentrations affected the nitrite reduction rates, a similar isotope enrichment factor (ε^15^N) for nitrite reduction to N_2_ of −4.5 ± 0.3‰ was determined. This ε^15^N value for nitrite reduction is on the low end of previously reported values. Chien *et al*.^45^ reported ε^15^N values between −33.2 and −2.9‰ in soil experiments. Martin and Casciotti (2016) calculated ε^15^N between −22 to −8‰ for reduction of nitrite associated with different marine strains.

During denitrification, a gradual decrease in NO_3_^−^ concentration is usually accompanied by a significant enrichment of ^15^N in the remaining nitrate^33^. The nitrogen isotope fractionation factors calculated in the current study for denitrification of nitrate (low and high nitrate treatments) of −21.1 and −24.4‰ are in good agreement with those found in the literature under similar conditions where denitrification occurs^58^. During nitrate reduction, the observed ^15^N enrichment of the remaining nitrate is caused by the preferential reduction of ^14^N-NO_3_^−^ and a consequent enrichment of ^14^N of the product and intermediate compound NO_2_^−^ during phase 1 of “net NO_2_^−^ production” as shown in Fig. 1. As a result, NO_2_^−^ concentrations increase and reach a maximum value during this phase 1 of denitrification (see Fig. 4a,b). During phase 2 or “net NO_2_^−^ reduction” (Fig. 1), the NO_2_^−^ concentration progressively decreases, leading to ^15^N enrichment of remaining NO_2_^−^ due to preferential reaction of ^14^N-NO_2_^−^. During phase 1, production and reduction of NO_2_^−^ take place simultaneously, however production occurs at a higher rate leading to an increase in nitrite concentrations. In phase 2, production and reduction of NO_2_^−^ also take place simultaneously, but since NO_2_^−^ reduction rates are higher than production rates, nitrite concentrations decrease.

Based on the N isotope enrichment factor (ε^15^Ν) measured for nitrite reduction alone (ε = −4.2‰) in the experiment with the low nitrite treatment, and combined with the ε^15^N of nitrate reduction (ε = −21‰), the model did not accurately predict the measured δ^15^N-NO_2_^−^ and constantly underestimated the measured δ^15^N-NO_2_^−^ value (Fig. 8). When the apparent N isotope enrichment factor (ε^15^Ν) of NO_2_^−^ reduction was changed to −10‰ in the model, an almost perfect fit between simulated and observed δ^15^N-NO_2_^−^ was achieved (Fig. 8a). A similar approach was used for the experiment with the high nitrate treatment with an N isotope enrichment factor varying between −10 and −15‰ to fit simulated and observed δ^15^N-NO_2_^−^ (Fig. 8b). For both low and high nitrate treatments, the apparent N isotopic enrichment factor of nitrite reduction required by the model to achieve a good fit with the observed data (ε^15^N = −10 and −15‰ respectively) is higher than the ε^15^N obtained from the experiments with nitrite as sole source of nitrogen (ε^15^N = −4.2 and −4.7‰ for low and high nitrite treatment respectively).

It is also interesting to note that the calculated rates in the model revealed that the nitrite production rate varies during nitrate reduction, as shown especially for the high nitrate treatment. The nitrogen isotope enrichment factor determined for experiments with nitrite as the sole electron acceptor was lower for both treatments (ε^15^N varied between −4.7 and −4.2‰) than suggested by the Isonitrite model, independent of the nitrite reduction rates or nitrite concentrations. The apparent nitrogen isotope fractionation of nitrite during nitrate reduction seemed to increase with increasing availability of nitrite. The amount of nitrite available for nitrite reductase during denitrification is variable and lower than in the experiments with nitrite addition. Hence, either the variable or the lower nitrite concentrations may explain the difference in nitrogen isotope enrichment factors compared with those calculated in the nitrite reduction experiment.

Nitrite reduction is mediated by two different types of periplasmic enzymes, the copper containing nitrite reductase (nirK or Cu-NIR) or the cytochrome cd1-containing nitrite reductase (nirS or Fe-NIR)^16^. During denitrification with nitrite as a sole electron acceptor, nitrite is directly used by one of these enzymes at the outer side of the cell wall. During the experiment with nitrate as electron acceptor, nitrite is produced in the cytoplasm and consumed within the periplasm. Nitrite concentrations as substrate are low compared to those in the case of nitrite addition. When nitrite is produced from the reduction of nitrate, the comparatively low amount of nitrite-nitrogen could be limiting and generate low N isotope fractionation. In addition to lower net concentrations of nitrite, nitrite is subject to transport upon reduction of nitrate, which may affect the extent of isotope fractionation^59^. Nitrate reduction can occur in the periplasm (via the enzyme Nap), but in most cases nitrate is reduced by the membrane bound nitrate reductase Nar. The membrane bound Nar reduces nitrate to nitrite in the cytoplasm, nitrite is then transported over the cell membrane via a transporter (nark or nasH) back to the periplasm where it is reduced further by either nirS or nirK^16^. The affinity with the transporter might thus generate nitrogen isotope fractionation. Combined transport and limiting nitrite concentrations could thus be responsible for higher nitrogen isotope fractionation during nitrate reduction as opposed to experiments were nitrite was the sole electron acceptor.

### O isotope fractionation during nitrate and nitrite reduction

During denitrification, a gradual decrease in NO_3_^−^ concentrations is usually accompanied by a significant enrichment of ^18^O in the remaining nitrate^44^. The oxygen isotope fractionation factors of −19.6 and −21.9‰ calculated in the current study are on the high end of what has been previously reported in the literature^42,44^.

In our study, the nitrogen and oxygen isotope fractionation during denitrification were in the same range (ε^15^N −21.1 to −24.4‰ and ε^18^O −19.6 to −21.9‰) for low and high nitrate treatments with a ratio of N and O isotope enrichment factors (^18^ε/^15^ε) observed in this study of 0.9 for low and high nitrate treatments. This is in agreement with results from a number of recent isotopic denitrification studies where the observed ^18^ε/^15^ε ratio was close to 1.0^50,60–62^. The role of the different nitrate reductases from various bacterial strains has recently been discussed potentially explaining the large range of ε^15^N values observed during denitrification in marine sediments^42^. Moreover, Frey *et al*.^63^ showed with pure cultures that the extent of N isotope fractionation during denitrification may depend on the different nitrate reduction enzymes (periplasmic Nap versus membrane bound Nar).

In the experiments with nitrite as sole source of nitrogen, δ^18^O-NO_2_^−^ remained constant during the nitrite reduction experiments. This is clear evidence for rapid oxygen isotope exchange between H_2_O and NO_2_^−^ under experimental conditions keeping the δ^18^O-NO_2_^−^ constant. Therefore, the observed oxygen isotope enrichment for denitrification of −21‰, as revealed in experimental low and high nitrate treatments, appears to be entirely associated with reduction of nitrate to nitrite.

The Isonitrite model was used to obtain a lower limit for the kinetics of oxygen isotope exchange between NO_2_^−^ and H_2_O. Kinetics of this exchange were estimated by ensuring that the oxygen isotope fractionation associated with the reduction of NO_3_^−^ and NO_2_^−^ was not able to deviate the δ^18^O-NO_2_^−^ from its equilibrium value with water having a δ^18^O-H_2_O value of −10‰. Repeated model runs varying the kinetic constant of the oxygen isotope exchange between NO_2_^−^ and H_2_O (Eq. 21 in Methods) suggest that this kinetic constant must be greater than 5 * 10^−10^ µM s^−1^ in order for δ^18^O-NO_2_^−^ to not deviate from its equilibrium value with water near 0‰ (Fig. 8). The fact that the δ^18^O-NO_2_^−^ remained constant despite that NO_2_^−^ production and consumption probably fractionate oxygen isotope ratios considerably suggests that the kinetic isotope constant for O isotope exchange between nitrite and water represented in Eq. (21) is greater than 3 * 10^−8^ µM^−1^ min^−1^.

In the absence of published values of the kinetic constant of oxygen isotope exchange k^+^ (reaction 17), it was compared with the chemical kinetic constant of NO_3_^−^ reduction k_NO3_ (Eq. 7). In order to do this comparison, the units of k^+^ (µM^−1^ min^−1^) were converted into the units of k_NO3_ (min^−1^) by considering relation 18 and multiplying k^+^ (µM^−1^ min^−1^) by the concentration of H_2_^16^O (55 × 10^6^ µM). A k^+^ constant of 1.65 min^−1^ was obtained which is 5 orders of magnitude higher than the k_NO3_ (4.2 × 10^−5^ min^−1^). It appears thus that oxygen isotope exchange in our experiment is very rapid compared to the microbial mediated reduction of NO_3_^−^. Previous studies hypothesized that isotopic exchange can occur between the oxygen of water and the oxygen of nitrite over long time periods^41,43,53^. Our study confirms rapid oxygen isotope exchange between the intermediate nitrite and water. Due to the fast change of the δ^18^O-NO_2_^−^, it appears that isotope variations are driven not by physical parameters but are caused by bacteria activity.

## Conclusions

This study reports the N and O isotope enrichment factors associated with the multi-step biogeochemical process of denitrification. Isotope measurements and calculation of isotope fractionation based on the Isonitrite model provide insights into the apparent isotopic fractionation of nitrite during denitrification. Due to rapid isotopic exchange between the oxygen of nitrite and water, δ^18^O-NO_2_^−^ changed immediately and remained stable during nitrate reduction. Model calculations also confirmed rapid oxygen isotope equilibrium exchange between water and nitrite with a kinetic isotope constant for O isotope exchange of >5 * 10^−2^ kg mol^−1^ s^−1^.

Based on N and O isotope enrichment factors obtained from batch experiments, model simulations revealed an apparent nitrogen isotope enrichment factor for nitrite reduction (of −10‰ and −15‰) higher than those calculated for the experiments (−4.1‰ and −4.7‰) with nitrite as initial source of nitrogen with two different concentrations. The reduction of nitrate seems to drive the change of the nitrogen isotope fractionation factor affecting nitrite reduction. We hypothesize that this difference is due to variations of the amount of available nitrite. This suggests that the amount of available nitrite and transport determine the extent of nitrogen isotope fractionation during the process of denitrification.

## Methods

### Experimental setup and materials

In order to determine the variations in nitrogen and oxygen isotope ratios, N and O isotopic enrichment factors for nitrite reduction were determined under denitrifying conditions (in the absence of oxygen) with NO_2_^−^ as the sole electron acceptor in a laboratory batch incubation experiment. Subsequently, the same experimental approach was used with NO_3_^−^ as electron acceptor in order to determinate the N (ε^15^N) and O (ε^18^O) isotope enrichment during denitrification of NO_3_^−^. For both experiments, two treatments at the low (101 µM for nitrite and 97 µM for nitrate) and high end (614 µM for nitrite and 508 µM for nitrate) of concentrations measured in surface waters were used. Subsequently, a non-steady state model was developed to simulate the changes in concentrations and N and O isotope ratios of nitrate and nitrite tracing kinetics and isotope variations during reduction of nitrate concentrations associated with initial net production of nitrite and subsequent net reduction of nitrite.

### Batch experiments

Muddy organic sediments were collected from the Morbras Stream, La Queue en Brie, a tributary of the Marne River in the Seine watershed upstream of Paris (France). Sediments from this stream had been used in a preliminary test and showed significant nitrate reduction potential (data not shown). The land use in the upstream catchment is mainly crops. The sediment was homogenized in the laboratory prior to the experiments. Contents and isotopic compositions ot total organic carbon and total nitrogen were determined by EA-IRMS (C_org_ = 8.6%; N_org_ = 0.8%; C/N = 14.9; δ^13^C_org_ = −24.6‰; δ^15^N_total_ = 2.9‰). Thirty grams of sediment were weighed in 300 ml glass bottles and 280 mL of nitrite or nitrate in milliQ water was added to create a sediment slurry. The slurries were flushed with He for twenty minutes to achieve anoxic conditions prior to crimp capping the bottles with a septum and a ring. The solutions in the first set of experiments contained 101 and 614 µM of nitrite derived from KNO_2_ (low and high nitrite treatments), and the second set of experiments contained 97 and 508 µM of nitrate derived from KNO_3_ (low and high nitrate treatments). Prior to addition, the salts were dissolved in milliQ water with a δ^18^O-H_2_O value of −10‰. Bottles were incubated under continuous agitation in order to maintain sediments in suspension and optimize denitrification (Sebilo *et al*. 2003) in the dark at 20 °C for 270 and 330 minutes for low and high nitrite treatments and for 180 and 420 minutes for low and high nitrate treatments, respectively. For each treatment, 14 similar slurries were prepared and considered homogeneous at the beginning of the experiment. Every 15 to 30 minutes for each treatment, a bottle was sacrificed and the slurry was centrifuged at 4000 rpm for 15 minutes. The supernatant was then filtered first through a 0.45μm glass microfiber filters (GFF-Whatman) and subsequently through a 0.2 µm nylon membrane (Whatman) to remove all sediment. Solutions were immediately preserved with mercuric chloride (0.3 ml at 5%) to avoid any residual transformation of the dissolved compounds in the solution. An aliquot of this solution was used to determine nitrite and nitrate concentrations. Ammonium concentrations were determined at the beginning and at the end of the experiments to check the ammonium budget. As ammonium consumption due to nitrification can be excluded under anoxic conditions, ammonium production should be only caused by ammonification (degradation of organic matter) or DNRA. Ammonium concentrations were below the limit of detection for all analyzed samples. Another aliquot of the solution was frozen and stored for subsequent N and O isotope analysis on nitrite and nitrate.

### Nutrient concentration analyses

Nutrient concentrations were determined by colorimetry. Nitrate concentrations were determined using the colorimetric hydrazine method. Nitrate was converted to nitrite with hydrazine. Then nitrite reacted with sulphanilamide and N-1-naphthylethylenediamine dihydrochloride. Nitrite concentrations were directly measured by colorimetry by using the same reactants. Ammonium reacted with sodium nitroprussiate and sodium hydroxide. The absorbance of coloration was determined at 540 nm and 660 nm (Gallery Water ThermoFisher Scientific) to obtain nitrite or nitrate and ammonium concentrations respectively with a precision of the measurement ±0.3 µM.

### Isotope ratio measurements

Nitrogen and oxygen isotope ratios of nitrate and nitrite were determined separately following a modified protocol of McIlvin and Altabet^64^. Nitrate was reduced to nitrite in an activated column of cadmium. Yields of nitrate reduction were calculated using concentration measurements with a yield of reduction >95%.

When both nitrite and nitrate concentrations were not negligible, the liquid samples were diluted with a salted buffer to obtain a concentration of 20 µM of NO_2_^−^ at the column outlet, which is composed of reduced NO_3_^−^ and the initial NO_2_^−^ (called later NO_x_^−^). Subsequently, the NO_2_^−^ (i.e. NO_x_^−^ and NO_2_^−^) was further reduced to nitrous oxide by sodium azide solution. To achieve this, each sample was diluted with a salt solution (NaCl = 35.5 g l^−1^) to obtain 1 µM of NO_2_^−^ concentration in 15 ml and transferred into a 20 ml glass vial. The vials were crimp-sealed with Teflon-backed silicone septa (IVA, 70220804) and aluminum caps. The sodium azide solution, which consists of a 40 ml mixture of 20 ml of azide (NaN_3_, 2 M) and 20 ml of acetic acid (C_2_H_4_O_2_, 20%), was flushed with He for 30 min to avoid any N_2_O pollution from the buffer. In order to reduce the NO_2_^−^ to N_2_O, 0.8 ml of azide solution was injected in each vial, and vials were placed in a water bath at 30 °C for one hour. The yield of conversion was better than 95%.

The isotope compositions of all N_2_O samples were measured with an isotope ratio mass spectrometer (IRMS, Delta Vplus, Thermo Scientific, Bremen, Germany) in continuous-flow mode with a purge-and-trap system coupled with a Finnigan GasBench II system (Thermo Scientific, Bremen, Germany). Results are reported in the internationally accepted delta notation in ‰ with respect to the standards air for δ^15^N and Vienna Standard Mean Ocean Water (V-SMOW) for δ^18^O, respectively. Nitrate and nitrite reference materials subject to the same analytical procedures were used to calibrate the isotopic composition of N_2_O (USGS34, δ^15^N = −1.8‰, δ^18^O = −27.9‰, USGS35, δ^15^N = +2.7‰, δ^18^O = +57.5‰ and USGS32, δ^15^N = +180‰, δ^18^O = +25.7‰ for nitrate standards; lab nitrite standards Lb1, δ^15^N = −63‰ and Lb2, δ^15^N = +2.7‰ for nitrite standards). The precision was ±0.3‰ for δ^15^N-NO_2_^−^ and ±0.5‰ for δ^18^O-NO_2_^−^.

Finally, using the isotopic compositions of NO_x_^−^ (NO_2_^−^ + NO_3_^−^) and NO_2_^−^ alone, the δ^15^N-NO_3_^−^ and δ^18^O-NO_3_^−^ were calculated as follows:

5 $${{\rm{\delta }}}^{15}{\rm{N}} \mbox{-} {{\rm{NO}}}_{3}^{-}=({{\rm{\delta }}}^{15}{\rm{N}}-{{\rm{NOx}}}^{-}\times [{{\rm{NOx}}}^{-}]-{{\rm{\delta }}}^{15}{\rm{N}}-{{\rm{NO}}}_{2}^{-}\times [{{\rm{NO}}}_{2}^{-}]/[{{\rm{NO}}}_{3}^{-}]$$

6 $${{\rm{\delta }}}^{18}{\rm{O}} \mbox{-} {{\rm{NO}}}_{3}^{-}=({{\rm{\delta }}}^{18}{\rm{O}}-{{\rm{NOx}}}^{-}\times [{{\rm{NOx}}}^{-}]-{{\rm{\delta }}}^{18}{\rm{O}}-{{\rm{NO}}}_{2}^{-}\times [{{\rm{NO}}}_{2}^{-}])/[{{\rm{NO}}}_{3}^{-}]$$

where [NO_3_^−^] and [NO_2_^−^] are the concentrations of NO_3_^−^ and NO_2_^−^, respectively.

Based on this calculation, the precision for δ^15^N-NO_3_^−^ was ±0.5‰ and ±0.8‰ for δ^18^O-NO_3_^−^.

### Determination of isotope enrichment factors

The nitrite experiment in a closed system without any renewal of substrate ensured Rayleigh conditions and therefore the N and O isotope enrichment factors (ε) were calculated using the following equations:

7 $${{\rm{\delta }}}^{15}{\rm{N}} \mbox{-} {{\rm{NO}}}_{2}^{-}({\rm{t}})={{\rm{\delta }}}^{15}{\rm{N}}-{{\rm{NO}}}_{2}^{-}({\rm{t}}0)+{\rm{\varepsilon }}\,\mathrm{ln}\,{\rm{f}}={{\rm{\delta }}}^{15}{\rm{N}}-{{\rm{NO}}}_{2}^{-}({\rm{t}}0)+{\rm{\varepsilon }}\,\mathrm{ln}\,C(t)/C(0)$$

and

8 $${{\rm{\delta }}}^{18}{\rm{O}} \mbox{-} {{\rm{NO}}}_{2}^{-}({\rm{t}})={{\rm{\delta }}}^{18}{\rm{O}}-{{\rm{NO}}}_{2}^{-}({\rm{t}}0)+{\rm{\varepsilon }}\,\mathrm{ln}\,{\rm{f}}={{\rm{\delta }}}^{18}{\rm{O}}-{{\rm{NO}}}_{2}^{-}({\rm{t}}0)+{\rm{\varepsilon }}\,\mathrm{ln}\,C(t)/C(0)$$

where δ^15^N and δ^18^O-NO_2_^−^ (t0) are the N and O isotope ratios of the substrate at time zero, and C0 and Ct are the concentrations of nitrite at time zero and t, respectively. This relationship was used to determine the N and O isotope enrichment factor of nitrite reduction by plotting measured values of δ^15^N(t) and δ^18^O(t) against ln C_t_/C_0_. The same approach was used to determine the N and O isotope enrichment factor for denitrification for the experiments where nitrate was the sole source of nitrogen.

### Isonitrite model

As nitrite is both a substrate and a product during denitrification of nitrate, Rayleigh conditions and equations are not applicable to determine the N and O isotope enrichment factor associated with the reduction of NO_2_^−^ when these two processes occur concurrently in a batch reactor. Although our batch experiments alone are sufficient to derive enrichment factors (ε) for the separately tested NO_3_^−^ = >NO_2_^−^ and NO_2_^−^ = >N_2_ steps, a model was developed to calculate the isotope enrichment factors when these processes occur simultaneously in a batch reactor. The model simulates the multi-step denitrification process and considers the presence of the intermediate product (NO_2_^−^) as well as the N and O isotopic compositions of nitrite and nitrate. The model is constrained with experimentally determined concentrations and N and O isotopic ratios of nitrite and nitrate. The model was used to estimate instantaneous isotopic enrichment factors associated with NO_3_^−^ reduction to NO_2_^−^ and NO_2_^−^ reduction to N_2_. Moreover, the model was used to investigate the kinetics of oxygen isotope exchange between water and NO_2_^−^.

The model considers that denitrification is a two-stage process:

9 $${{\rm{NO}}}_{3}^{-}+2{{\rm{e}}}^{-}+2{{\rm{H}}}^{+}\to {{\rm{NO}}}_{2}^{-}+{{\rm{H}}}_{2}{\rm{O}}$$

10 $${{\rm{NO}}}_{2}^{-}+3{{\rm{e}}}^{-}+4{{\rm{H}}}^{+}\to 1/2{{\rm{N}}}_{2}+2{{\rm{H}}}_{2}{\rm{O}}$$

The rate of these processes is computed as:

11 $${{\rm{R}}}_{{\rm{NO}}3}={{\rm{k}}}_{{\rm{NO}}3}\times {{\rm{NO}}}_{3}^{{\rm{nNO}}3}$$

and

12 $${{\rm{R}}}_{{\rm{NO}}2}={{\rm{k}}}_{{\rm{NO}}2}\times {{\rm{NO}}}_{2}^{{\rm{nNO}}2}$$

where NO_3_^−^ and NO_2_^−^ concentrations are expressed in µM, R_x_ (x = NO_3_^−^ or NO_2_^−^) is the nitrite production or reduction (reaction) rate in µM min^−1^, k_x_ is the kinetic constant in min^−1^ and nx is the reaction order.

To simulate both N and O isotope ratios simultaneously, three isotopic species must be considered for each chemical species, with the total chemical concentration being equal to the sum of the isotopic species:

13 $${{\rm{NO}}}_{3}={}^{14}{\rm{N}}_{16}{{\rm{O}}}_{3}+{}^{15}{\rm{N}}^{16}{{\rm{O}}}_{3}+{}^{14}{\rm{N}}^{18}{{\rm{O}}}_{3}$$

14 $${{\rm{NO}}}_{2}={}^{14}{\rm{N}}_{16}{{\rm{O}}}_{2}+{}^{16}{\rm{N}}^{15}{{\rm{O}}}_{2}+{}^{14}{\rm{N}}^{18}{{\rm{O}}}_{2}$$

With ^18^O_3_: ^18^O^16^O^16^O and ^18^O_2_: ^18^O^16^O.

From these isotopic species, the δ^15^N and the δ^18^O values of NO_2_^−^ and NO_3_^−^ can be calculated as follows (equations shown for NO_3_):

15 $${\delta }^{15}{N}_{N{O}_{3}}=(\frac{{}^{15}N{}^{16}O_{3}}{{}^{14}N{}^{16}O_{3}+{}^{14}N{}^{18}O_{3}}/{N}_{STD}-1)\times {10}^{3}$$

16 $${\delta }^{18}{O}_{N{O}_{3}}=(\frac{{}^{14}N{}^{18}O_{3}}{3\cdot ({}^{14}N{}^{16}O_{3}+{}^{15}N{}^{16}O_{3})+2\cdot ({}^{14}N{}^{18}O_{3})}/SMOW-1)\times {10}^{3}$$

where SMOW is the ^18^O/^16^O ratio in the SMOW standard and N_STD_ is the ^15^N/^14^N ratio of air.

The rate of reaction for NO_3_^−^ and NO_2_^−^ reduction for the different isotope species have to sum up to the rate of the total chemical species calculated with Eqs. 3 and 4. For example, for NO_3_^−^ reduction:

17 $${R}_{N{O}_{3}}={R}_{{}^{14}N{}^{16}O_{3}}+{R}_{{}^{15}N{}^{16}O_{3}}+{R}_{{}^{14}N{}^{18}O_{3}}$$

The expressions for the rates of the isotopic species are obtained by solving a system of three equations comprising equation 14 and equations 12 and 13 written in terms of rates rather than concentrations, and introducing isotopic enrichment factors for N and O isotopes (^15^ε_NO3_ and ^18^ε_NO3_, respectively):

18 $${R}_{{}^{14}N{}^{16}O_{3}}=\frac{{R}_{N{O}_{3}}\cdot ({10}^{6}-({10}^{3}+{\delta }^{18}{O}_{N{O}_{3}}+{}^{18}\varepsilon _{N{O}_{3}})\cdot (2\cdot {10}^{3}+3\cdot ({10}^{3}+{\delta }^{15}{N}_{N{O}_{3}}+{}^{15}\varepsilon _{N{O}_{3}})\cdot {N}_{STD})\cdot SMOW)}{({10}^{3}+({10}^{3}+{\delta }^{15}{N}_{N{O}_{3}}+{}^{15}\varepsilon _{N{O}_{3}})\cdot {N}_{STD})\cdot ({10}^{3}+({10}^{3}+{\delta }^{18}{O}_{N{O}_{3}}+{}^{18}\varepsilon _{N{O}_{3}})\cdot SMOW)}$$

19 $${R}_{{}^{15}N{}^{16}O_{3}}=\frac{{R}_{N{O}_{3}}\cdot ({10}^{3}+{\delta }^{15}{N}_{N{O}_{3}}+{}^{15}\varepsilon _{N{O}_{3}})\cdot {N}_{STD}}{{10}^{3}+({10}^{3}+{\delta }^{15}{N}_{N{O}_{3}}+{}^{15}\varepsilon _{N{O}_{3}})\cdot {N}_{STD}}$$

20 $${R}_{{}^{14}N{}^{18}O_{3}}=\frac{3\cdot {R}_{N{O}_{3}}\cdot ({10}^{3}+{\delta }^{18}{O}_{N{O}_{3}}+{}^{18}\varepsilon _{N{O}_{3}})\cdot SMOW}{{10}^{3}+({10}^{3}+{\delta }^{18}{O}_{N{O}_{3}}+{}^{18}\varepsilon _{N{O}_{3}})\cdot SMOW}$$

Exchange of oxygen isotopes between NO_2_^−^ and H_2_O takes place according to the following isotopic exchange reaction:

21 $$N{}^{18}O_{2}+{H}_{2}{}^{16}O\underset{{k}^{-}}{\overset{{k}^{+}}{\leftrightarrow }}N{}^{16}O_{2}+{H}_{2}{}^{18}O$$

The forward and backwards rates of this reaction are calculated as:

22 $${R}_{+}^{N{O}_{2}\leftrightarrow {H}_{2}O}={k}^{+}\cdot N{}^{18}O_{2}\cdot {H}_{2}{}^{16}O$$

23 $${R}_{-}^{N{O}_{2}\leftrightarrow {H}_{2}O}={k}^{-}\cdot N{}^{16}O_{2}\cdot {H}_{2}{}^{18}O$$

where N^16^O_2_ = ^14^N^16^O_2_ + ^15^N^16^O_2_, the rates are in µM min^−1^, and the forwards (k^+^) and backwards (k^−^) kinetic constants (in µM^−1^ min^−1^) are related to the equilibrium constant of the isotope exchange reaction a follows:

24 $${K}_{EQ}^{N{O}_{2}\leftrightarrow {H}_{2}O}=\frac{{k}^{-}}{{k}^{+}}$$

where the equilibrium constant for the isotope exchange reaction is obtained from the equilibrium concentrations of the species involved in the oxygen isotope exchange:

25 $${K}_{EQ}^{N{O}_{2}\leftrightarrow {H}_{2}O}=\frac{({[{}^{14}N{}^{16}O_{3}]}_{i}+{[{}^{15}N{}^{16}O_{3}]}_{i})\cdot {[{H}_{2}{}^{18}O]}_{i}}{{[{}^{14}N{}^{18}O_{3}]}_{i}\cdot {[{H}_{2}{}^{16}O]}_{i}}$$

where the subscript *i* refers to the initial, equilibrium concentration of isotopic species.

The concentrations of the six isotopic model species in time are calculated by integrating the following system of six coupled differential equations starting from initial chemical and isotopic equilibrium conditions:

26 $$\frac{\partial ({}^{14}N{}^{16}O_{3})}{\partial t}=-{R}_{{}^{14}N{}^{16}O_{3}}$$

27 $$\frac{\partial ({}^{15}N{}^{16}O_{3})}{\partial t}=-{R}_{{}^{15}N{}^{16}O_{3}}$$

28 $$\frac{\partial ({}^{14}N{}^{18}O_{3})}{\partial t}=-{R}_{{}^{14}N{}^{18}O_{3}}$$

29 $$\begin{array}{rcl}\frac{\partial ({}^{14}N{}^{16}O_{2})}{\partial t} & = & +{R}_{{}^{14}N{}^{16}O_{3}}-{R}_{{}^{14}N{}^{16}O_{2}}+\,\frac{{}^{14}N{}^{16}O_{2}}{{}^{15}N{}^{16}O_{2}+{}^{14}N{}^{16}O_{2}}\cdot {R}_{+}^{N{O}_{2}\leftrightarrow {H}_{2}O}\\  &  & -\,\frac{{}^{14}N{}^{16}O_{2}}{{}^{15}N{}^{16}O_{2}+{}^{14}N{}^{16}O_{2}}\cdot {R}_{-}^{N{O}_{2}\leftrightarrow {H}_{2}O}\end{array}$$

30 $$\begin{array}{rcl}\frac{\partial ({}^{15}N{}^{16}O_{2})}{\partial t} & = & +{R}_{{}^{15}N{}^{16}O_{3}}-{R}_{{}^{15}N{}^{16}O_{2}}+\,\frac{{}^{15}N{}^{16}O_{2}}{{}^{15}N{}^{16}O_{2}+{}^{14}N{}^{16}O_{2}}\cdot {R}_{+}^{N{O}_{2}\leftrightarrow {H}_{2}O}\\  &  & -\,\frac{{}^{15}N{}^{16}O_{2}}{{}^{15}N{}^{16}O_{2}+{}^{14}N{}^{16}O_{2}}\cdot {R}_{-}^{N{O}_{2}\leftrightarrow {H}_{2}O}\end{array}$$

31 $$\frac{\partial ({}^{14}N{}^{18}O_{2})}{\partial t}=+{R}_{{}^{14}N{}^{18}O_{3}}-{R}_{{}^{14}N{}^{18}O_{2}}-{R}_{+}^{N{O}_{2}\leftrightarrow {H}_{2}O}+{R}_{-}^{N{O}_{2}\leftrightarrow {H}_{2}O}$$

To summarise, known model input parameters are: (1) the simulation time (that corresponds to the duration of the experiment), (2) the initial concentrations of NO_3_^−^ and NO_2_^−^, (3) the oxygen and nitrogen isotope compositions of NO_3_^−^ and NO_2_^−^, and (4) the δ^18^O of water. Unknown (fitting) parameters are: (1) the kinetic constants for NO_2_^−^ production, for NO_2_^−^ consumption and for isotope exchange between NO_2_^−^ and H_2_O, and (2) the oxygen and nitrogen isotope fractionation factors in NO_2_^−^ production and NO_2_^−^ consumption.

The model was implemented with the software Mathematica version 9 (Wolfram Research). Model outputs are changes of nitrite and nitrate concentrations and nitrogen and oxygen isotope compositions for both NO_2_^−^ and NO_3_^−^.
